# Clinical usefulness and acceleratory effect of macrophage inhibitory cytokine-1 on biliary tract cancer: an experimental biomarker analysis

**DOI:** 10.1186/s12935-022-02668-x

**Published:** 2022-08-10

**Authors:** Mitsuru Sugimoto, Rei Suzuki, Yoshihiro Nozawa, Tadayuki Takagi, Naoki Konno, Hiroyuki Asama, Yuki Sato, Hiroki Irie, Jun Nakamura, Mika Takasumi, Minami Hashimoto, Tsunetaka Kato, Ryoichiro Kobashi, Osamu Suzuki, Yuko Hashimoto, Takuto Hikichi, Hiromasa Ohira

**Affiliations:** 1 Department of Gastroenterology, School of Medicine, Fukushima Medical University, Fukushima, Japan; 2 Department of Pathology, Shirakawa Kousei General Hospital, Shirakawa, Japan; 3 Department of Endoscopy, Fukushima Medical University Hospital, Fukushima, Japan; 4 Department of Diagnostic Pathology, School of Medicine, Fukushima Medical University, Fukushima, Japan

**Keywords:** Apoptosis, Biliary tract cancer, Macrophage inhibitory cytokine-1, M30, Diagnostic marker

## Abstract

**Background:**

Biliary tract cancer (BTC) has a poor prognosis; therefore, useful biomarkers and treatments are needed. Serum levels of macrophage inhibitory cytokine-1 (MIC-1), a member of the TGF-β superfamily, are elevated in patients with pancreaticobiliary cancers. However, the effect of MIC-1 on BTC is unknown. Therefore, we investigated the effect of MIC-1 on BTC and assessed whether MIC-1 is a biomarker of or therapeutic target for BTC.

**Methods:**

MIC-1 expression in BTC cells was determined by performing histological immunostaining, tissue microarray (TMA), western blotting, and reverse transcription PCR (RT–PCR). Cell culture experiments were performed to investigate the effect of MIC-1 on BTC cell lines (HuCCT-1 and TFK-1). The relationships between serum MIC-1 levels and either the disease state or the serum level of the apoptosis marker M30 were retrospectively verified in 118 patients with pancreaticobiliary disease (individuals with benign disease served as a control group, n = 62; BTC, n = 56). The most efficient diagnostic marker for BTC was also investigated.

**Results:**

MIC-1 expression was confirmed in BTC tissue specimens and was higher in BTC cells than in normal bile duct epithelial cells, as determined using TMA, western blotting and RT–PCR. In cell culture experiments, MIC-1 increased BTC cell proliferation and invasion by preventing apoptosis and inhibited the effect of gemcitabine. In serum analyses, serum MIC-1 levels showed a positive correlation with BTC progression and serum M30 levels. The ability to diagnose BTC at an early stage or at all stages was improved using the combination of MIC-1 and M30. The overall survival was significantly longer in BTC patients with serum MIC-1 < the median than in BTC patients with serum MIC-1 ≥ the median.

**Conclusions:**

MIC-1 is a useful diagnostic and prognostic biomarker and might be a potential therapeutic target for BTC.

## Background

At the time of diagnosis, BTC has often advanced to affect multiple biliary tracts. In many patients, BTC is unresectable and has a poor prognosis [1, 2]. Chemotherapy is commonly administered to patients with unresectable BTC; however, the most effective regimen confers an overall survival of only 11–15 months [3–7]. Additionally, no specific serum tumour markers are available to diagnose or predict the prognosis of BTC [8]. Although the sensitivity of CA19-9 for diagnosing BTC was reported to be 71–72% [9, 10], an elevated CA19-9 level is observed in patients with nonmalignant obstructive jaundice [11, 12]. Therefore, an appropriate diagnostic or predictive prognostic marker for BTC is needed.

Macrophage inhibitory cytokine-1 (MIC-1) is a member of the TGF-β superfamily [13]. Elevated serum MIC-1 levels have been observed in patients with several inflammatory diseases and malignant tumours [14]. In addition, serum MIC-1 levels are markedly higher in patients with pancreatic cancer or BTC than in those with pancreaticobiliary inflammatory diseases or other cancers [15–18]. Although many reports on pancreatic cancer and MIC-1 have been published [19–25], few studies have investigated BTC and MIC-1. A previous study reported that biliary MIC-1 efficiently diagnosed early BTC [18]. However, the process of bile collection to measure biliary MIC-1 is invasive; thus, a less invasive diagnostic method is desired.

The effect of MIC-1 on each tumour type is different. In BTC, researchers have not determined whether MIC-1 enhances or prevents carcinogenesis. This study aimed to clarify the involvement of MIC-1 in BTC and the efficacy of MIC-1 as a noninvasive biomarker or therapeutic target for BTC.

## Methods

This study was approved by the Ethics Committee of Fukushima Medical University (Approval Number: 2387). All procedures were performed according to the manufacturers’ instructions.

### Immunostaining of surgical specimens and tissue microarray (TMA)

BTC specimens were immunostained for MIC-1. An anti-GDF-15/MIC-1 polyclonal antibody (Bioss Antibodies Inc., Massachusetts, USA) was used for MIC-1 immunostaining according to the manufacturer’s protocol. The microscope slides were deparaffinized and boiled in 0.01 M sodium citrate buffer (pH 6) at 100 °C for 15–20 min for antigen retrieval. The slides were treated with 3% hydrogen peroxide for 30 min to block endogenous peroxidase activity. Immunostaining was performed using the abovementioned antibody (dilution 1:1000).

BTC specimens were acquired during surgery. Samples from the patients whose surgical specimens were immunostained were included in the in vivo experiments described below. All patients provided written informed consent to participate in this study.

A TMA containing 54 BTC tissues and 25 normal bile duct tissues was purchased from Provitro (catalogue number: 401 22,078, Berlin, Germany). The TMA slides were deparaffinized, rehydrated, and immunostained in the same method used for the immunostaining of surgical specimens. The immunostaining intensity was defined as follows (0: negative, 1: weak, 2: moderate, and 3: strong). Finally, the intensity score was calculated as follows: (1 x % weakly positive cells) + (2 x % moderately positive cells) + (3 x % strongly positive cells). These TMA evaluations were performed at × 400 magnification. The maximum intensity score was defined as 300 [26, 27].

### Cell culture

A commercially available bile duct epithelial cell line (MMNK-1) and BTC cell lines (HuCCT-1 and TFK-1) were used in this study. As described above, BTC sometimes advances to extensively affect multiple biliary tracts. Therefore, TFK-1 cells were used as extrahepatic BTC cells, and HuCCT-1 cells were used as intrahepatic BTC cells. MMNK-1 and HuCCT-1 cells were purchased from JCRB Cell Bank, whereas TFK-1 cells were purchased from the Cell Resource Center for Biochemical Research, Cell Bank, Tohoku University. The MMNK-1 cell line was established from the liver by Maruyama et al*.* [28], the HuCCT-1 cell line was established from malignant ascites by Miyagiwa et al*.* [29], and the TFK-1 cell line was established by Saijyo et al*.* [30] from a common bile duct cancer specimen.

MMNK-1 cells were cultured in DMEM supplemented with 5% foetal bovine serum (FBS) and 1% penicillin–streptomycin in a humidified environment with 5% CO_2_ at 37 °C. The BTC cell lines were cultured in RPMI medium supplemented with 10% FBS and 1% penicillin–streptomycin in a humidified environment with 5% CO_2_ at 37 °C. Each cell culture experiment was performed in triplicate.

### Western blotting

Western blotting was performed using a previously described protocol [31]. The seeded cells were removed from the culture dishes and centrifuged, and the resulting cell pellets were lysed in RIPA buffer (Thermo Fisher Scientific, Waltham, MA, USA) supplemented with a protease inhibitor cocktail (Thermo Fisher Scientific). The lysates were mixed with sample buffer (Sigma, St. Louis, MO, USA) at a 1:1 ratio and then resolved on SDS–PAGE gels. After electrophoresis, the proteins were transferred to a PVDF membrane; the membrane was incubated with the primary antibody against GDF15 (1:1000, rabbit monoclonal antibody, no. 8479; Cell Signaling Technology, Beverly, MA, USA) at 4 °C overnight, anti-rabbit IgG secondary antibody (1:1000, no. 7074; Cell Signaling Technology) for one hour at room temperature, and an anti-β-actin polyclonal antibody (MBL, Tokyo, Japan) for one hour at room temperature. Blots were visualized using an Amersham Imager 600 (Cytiva, Tokyo, Japan) and Immobilon Clasico (Sigma–Aldrich, St. Louis, MO, USA). The immunoblots were evaluated by quantifying the band intensity using ImageJ software.

### Reverse transcription PCR (RT–PCR)

Total RNA was extracted from MMNK-1, HuCCT-1, and TFK-1 cells using an RNeasy^®^ Mini Kit (Qiagen, Hilden, Germany). The RNA concentration was measured using a NanoDrop 2000 instrument (Thermo Fisher Scientific). The cDNA templates were synthesized using an iScript Advanced cDNA Synthesis Kit for RT–PCR (Bio-Rad, Hercules, CA, USA) and a C1000 Touch™ Thermal Cycler (Bio-Rad). Real-time PCR was performed using QuantStudio 3 (Thermo Fisher Scientific) and TaqMan Gene Expression Assays (Thermo Fisher Scientific) with the following primers: GDF15 Hs00171132_m1 (catalogue no. 4331182) and GAPDH Hs02786624_g1 (catalogue no. 4331182). The mix was heated at 95 °C for 20 s and then amplified at 95 °C for 1 s and 60 °C for 20 s in 40 cycles. The Ct (threshold value) of each sample was obtained according to the threshold cycles with the software provided with the equipment, and the relative expression of the MIC-1 mRNA was renormalized to the expression of the GAPDH mRNA.

### Recombinant MIC-1 protein

Recombinant human GDF15/MIC-1 (PeproTech, Cranbury, NJ, USA) was used in all cell culture experiments. In cell proliferation assays, MIC-1 was used at concentrations recommended by both the manufacturer and previous reports [32, 33]. According to the manufacturer, MIC-1 was used at a concentration of 200 ng/ml. Therefore, the concentration of MIC-1 was diluted from 200 ng/ml to 100, 50, 25, 12.5, and 6.25 ng/ml, which were similar to the concentrations used in previous reports. In the other assays, the lowest effective concentration in the cell proliferation assay was applied.

### Cell proliferation assay

HuCCT-1 and TFK-1 cells were seeded in 96-well plates at a density of 5 × 10^3^ cells per well with 100 µl of medium supplemented with 10% FBS. After the plates were incubated overnight, MIC-1 solution (0, 6.25, 12.5, 25, 50, 100, or 200 ng/ml) was added to the cells and incubated for 3–7 days, after which Cell Counting Kit-8 (CCK-8) assays (Dojindo, Kumamoto, Japan) were performed. The absorbance was measured at 450 nm using a Benchmark Plus microplate spectrophotometer (Bio-Rad Laboratories, Hercules, CA).

### Cell invasion assay

Cell invasion assays were performed using 24-well Corning BioCoat™ Matrigel Invasion Chambers (Corning, New York, NY, USA) according to the manufacturer’s protocol. Five hundred microlitres of medium containing 10% FBS and MIC-1 were added to the lower chamber, while HuCCT-1 and TFK-1 cells (2.0 × 10^5^ cells/well) were added to the upper chamber in 500 µl of serum-free medium. After 22 h of incubation, cells that did not invade through the membrane were removed from the upper chamber using a cotton swab. The invaded cells were fixed with 4% paraformaldehyde for 20 min and stained with a 1% crystal violet solution for 30 min at room temperature. Three different fields were photographed using a BX41-13 microscope (Olympus, Tokyo, Japan) at 200 × magnification, and invasive cells were counted.

### Apoptosis assay

Apoptosis assays were performed using a Caspase-3/7 Fluorescence Assay Kit (Cayman Chemical, Michigan, USA). HuCCT-1 (2.0 × 10^5^ cells/well) and TFK-1 (3.0 × 10^5^ cells/well) cells were seeded in 100 µl of medium supplemented with 10% FBS and incubated overnight. After the medium was removed, the cells were exposed to the MIC-1 solution for three hours. Next, the cells were lysed with cell lysis buffer, after which the levels of active caspase 3 or caspase 7 were measured at an excitation wavelength of 485 nm and an emission wavelength of 535 nm using a Varioskan LUX multimode microplate reader (Thermo Fisher Scientific, MA, USA).

### Anticancer drug sensitivity assay

The CCK-8 assay (Dojindo) was performed to determine the effects of MIC-1 and gemcitabine on cell viability and proliferation. First, the effective gemcitabine concentration was determined. HuCCT-1 and TFK-1 cells were seeded in a 96-well plate at a density of 5 × 10^3^ cells/well in medium and incubated for 24 h. After the medium was removed, gemcitabine (Selleck, Houston, TX, USA) was added to each well at the indicated concentration (0, 1, 10, or 100 nmol/L), and the plates were incubated for 7 days. Then, CCK-8 solution was added to each well, and the absorbance was measured at 450 nm using a Benchmark Plus microplate spectrophotometer (Bio-Rad Laboratories). Second, the effect of MIC-1 on gemcitabine toxicity was investigated. Cells were seeded and incubated as described above. The cells were exposed to effective concentrations of gemcitabine and MIC-1 for 7 days. The absorbance was measured after the addition of the CCK-8 solution.

### Serum analyses

Serum MIC-1 levels were measured in patients with biliary tract diseases. Serum levels of M30, a selective apoptosis marker, were also measured. The M30 antibody detects cytokeratin-18 fragments that are cleaved during apoptosis, and the presence of M30 in bile duct epithelial and BTC cells was previously reported [34, 35]. The relationships between MIC-1 and both pathology and serum levels of an apoptotic marker were investigated.

### Patients

One hundred eighteen patients with biliary disease who were treated at Fukushima Medical University over a 4-year period were enrolled in this study. The median age of these patients was 72 years. The age range of the patients was 45–101 years. Among these patients, 56 had BTC (intrahepatic BTC: 6, peri-hilar BTC: 23, and extrahepatic BTC: 27), and 62 had benign biliary diseases and participated as control subjects [47 patients with common bile duct (CBD) stones and 15 patients with benign biliary stricture (5 with autoimmune pancreatitis (AIP), 3 with pancreatitis of unknown origin, 3 with chronic pancreatitis, 2 with primary sclerosing cholangitis, 1 with ampullary inflammation, and 1 with intraductal papillary neoplasm (IPMN)]. Patients were diagnosed with BTC using bile/brush cytology, endoscopic biopsy, endoscopic ultrasonography-guided fine needle aspiration, or surgery. Class IV or V cytology was defined as malignant disease. AIP was diagnosed according to the 2010 International Consensus Diagnostic Criteria [36]. The patient with IPMN presented no worrisome features or high-risk stigmata, as defined by international guidelines [37], and exhibited no signs of malignancy for more than 1 year of imaging follow-up. Cancer progression was evaluated according to the Union for International Cancer Control (UICC) classification, ver. 8 [38]. All patients provided written informed consent for participation in this study.

### Measurement of serum MIC-1 and M30 levels

Frozen sera were thawed at room temperature. Serum MIC-1 levels were measured using a Quantikine ELISA Human GDF-15 immunoassay kit (R & D Systems, Minneapolis, MN, United States), whereas serum M30 levels were measured using an M30 Apoptosense ELISA kit (VLVbio AB, Nacka, Sweden). Both kits were used according to the manufacturer’s instructions.

### Examination items

Serum MIC-1 and M30 levels in patients with BTC were compared with those in control subjects. Additionally, patient characteristics (age and sex) and the levels of several serum markers (AST, ALT, CRP, and CA19-9) were compared. The relationships between serum MIC-1, ALT and M30 levels and cancer progression were investigated. Additionally, the most efficient diagnostic marker for BTC and early BTC (stage I or II) was investigated. The most efficient diagnostic marker was investigated in patients in whom all biomarkers (CA19-9, MIC-1, and M30) were measured. Finally, the prognostic predictive capacities of MIC-1 and M30 for BTC patients were investigated. For the prognostic assessment, disease-free survival (DFS) and overall survival (OS) were evaluated. DFS was examined in patients who underwent surgery and was defined as the period from the date of surgery to the date of recurrence or the last follow-up date. BTC recurrence was confirmed by CT. OS was defined as the period from the date of pretreatment blood sampling for biomarkers to the date of death or the last follow-up date.

### Statistical analysis

An unpaired Student’s t test was used to compare data from cell culture experiments. Because the number of subjects was sufficient, an unpaired Student’s or Welch’s t test was also used to compare continuous variables. The Mann–Whitney U test was used to compare continuous variables that did not display a normal distribution. A chi-square test was used to compare categorical variables. Spearman’s rank correlation coefficient was calculated to investigate the correlations between two values. The ability to diagnose BTC using various biomarkers was compared by constructing receiver operating characteristic (ROC) curves. The prognostic assessment was analysed by the log-rank test. All statistical analyses were performed using IBM SPSS Statistics (IBM Corp., Armonk, NY, USA) and the EZR platform (Saitama Medical Center, Jichi Medical University, Saitama, Japan), which is a graphical user interface for R (The R Foundation for Statistical Computing, Vienna, Austria). More precisely, EZR is a modified version of the R commander that was designed to perform functions frequently used in biostatistics [39].

## Results

### MIC-1 expression determined using immunostaining, western blotting, and RT–PCR

**Fig. 1:**
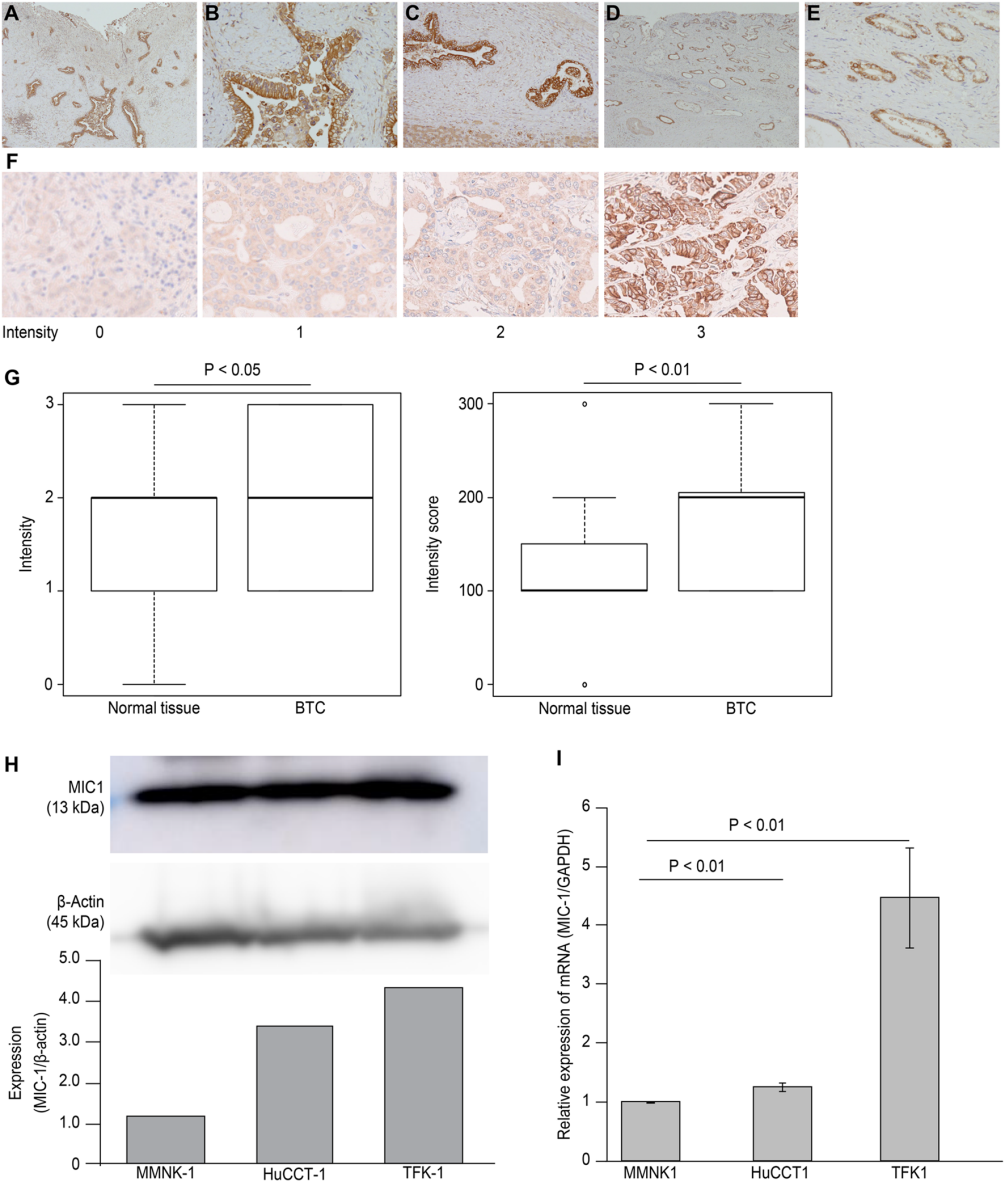
Histological immunostaining, TMA, western blot and RT–PCR analyses of MIC-1 expression. MIC-1 immunostaining was performed on tumour specimens from two patients with BTC (magnification: **A** × 20, **B** × 200, **D** × 20, **E** × 100). MIC-1 expression was observed in specimens from both patients. **C** MIC-1 expression was also observed in normal bile duct epithelial cells from the first patient (magnification: × 100). **F** The intensity of TMA immunostaining is shown (0: none, 1: weakly positive, 2: moderately positive, and 3: strongly positive) (magnification × 200). **G** MIC-1 expression was higher in BTC tissues than in normal tissues in the TMA. **H** MIC-1 was more expressed at higher levels in tumour cell lines (HuCCT-1 and TFK-1) than in a normal bile duct epithelial cell line (MMNK-1), as determined using western blotting. **I** MIC-1 expression was also more frequently detected in tumour cell lines using RT–PCR

MIC-1 immunostaining was performed on two surgical specimens (Fig. 1A–E), and MIC-1 expression was observed in the cytoplasm of BTC cells (Fig. 1A, B, D, E) and normal bile duct epithelial cells (Fig. 1C). The intensity of TMA immunostaining is shown in Fig. 1F. The intensity was significantly higher in BTC tissues than in normal tissues (*p* = 0.039) (Fig. 1G). The intensity score was also significantly higher in BTC tissues than in normal tissues (*p* < 0.01). Higher MIC-1 expression was detected in BTC cells (HuCCT-1 and TFK-1) than in normal bile duct epithelial cells (MMNK-1) using western blotting (Fig. 1H) and RT–PCR (Fig. 1I).

### Cell culture experiments

#### Cell proliferation assay

**Fig. 2:**
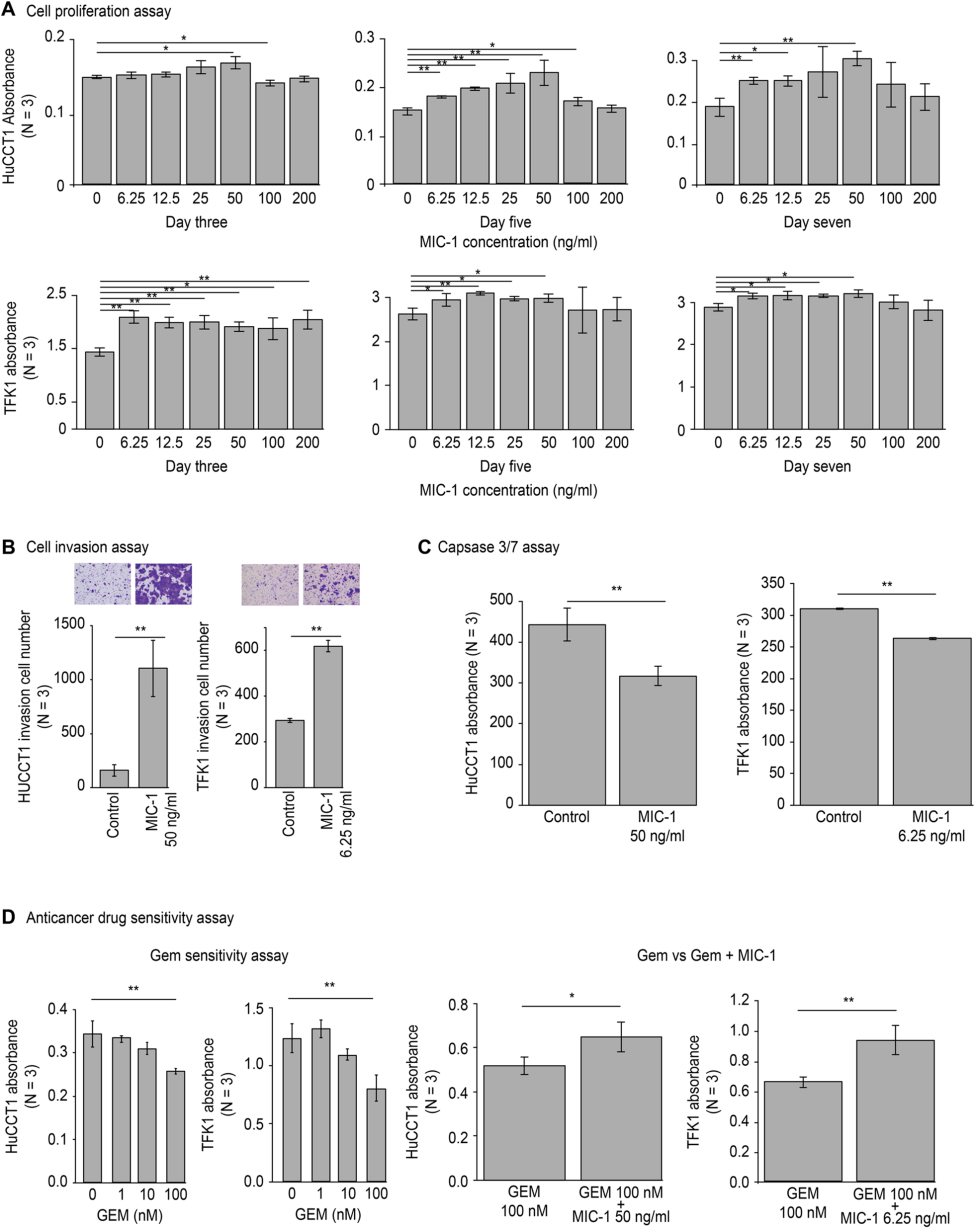
Results of the cell culture experiments (n = 3). **A** Cell proliferation assay. The proliferation of both BTC cell lines was significantly accelerated by MIC-1. At three days, the minimum effective concentration of MIC-1 was 50 ng/ml in HuCCT-1 cells and 6.25 ng/ml in TFK-1 cells. These concentrations were applied in the other cell culture experiments. **B** Cell invasion assay. BTC cell invasion was accelerated by MIC-1. **C** Apoptosis assay. A higher level of apoptosis was observed in cell lines not treated with MIC-1 than in those treated with MIC-1. **D** Anticancer drug sensitivity assay. The effective concentration of GEM was 100 nM in both tumour cell lines (left two figures). MIC-1 inhibited the anticancer effect of GEM (right two figures). * *P* < 0.05 and ** *P* < 0.01

MIC-1 potentiated the proliferation of both HuCCT-1 and TFK-1 cells (Fig. 2A). After only three days, 50 ng/ml MIC-1 promoted the proliferation of HuCCT-1 cells, whereas 6.25 ng/ml MIC-1 increased TFK-1 cell proliferation. Therefore, in subsequent cell culture experiments, 50 ng/ml MIC-1 was used to treat HuCCT-1 cells, and 6.25 ng/ml MIC-1 was used to treat TFK-1 cells. On the other hand, 100 or 200 ng/ml MIC-1 did not result in significant BTC cell proliferation.

#### Cell invasion assay

Cell invasion was significantly greater in the BTC cells that were exposed in MIC-1 than in the controls (Fig. 2B). MIC-1 facilitated the invasion of both HuCCT-1 and TFK-1 cells.

#### Cell apoptosis assay

In caspase 3/7 assays, more apoptotic cells were observed in the control cells than in the BTC cells that were exposed in MIC-1 (Fig. 2C). MIC-1 suppressed apoptosis of both HuCCT-1 and TFK-1 cells.

#### Anticancer drug sensitivity assay

First, an appropriate gemcitabine concentration was investigated in both HuCCT-1 and TFK-1 cells. Gemcitabine at 100 nM reduced the number of HuCCT-1 and TFK-1 cells (Fig. 2D: left). MIC-1 inhibited this tumour-suppressive effect of gemcitabine in two BTC cell lines (Fig. 2D: right).

#### Serum analyses

Age, sex, and the levels of transaminases (AST and ALT), CRP, and CA19-9 were not significantly different between the control (benign disease) patients and patients with BTC (Table 1). Serum M30 and MIC-1 levels were significantly higher in patients with BTC than in controls (M30: 464.2 ± 305.7 vs. 212.5 ± 133.5 U/ml, *p* < 0.01; MIC-1: 379.0 ± 204.5 vs. 228.2 ± 149.2 × 10^–2^ ng/ml, *p* < 0.01).

**Table 1:** Comparison of clinical and demographic characteristics and serum markers in patients with benign disease (control) or BTC

	Control	BTC	*P* value
N	62	56	
Age, y, mean ± SD	72.3 ± 11.8	72.5 ± 8.4	0.93
Male/female, n	38/24	39/17	0.45
The location of BTC			
Intrahepatic		6	
Peri-hilar		23	
Extrahepatic		27	
UICC stage			
I		16	
II		14	
III		12	
IV		14	
AST, U/L, mean ± SD	115.7 ± 203.0	126.5 ± 200.4	0.77
ALT, U/L, mean ± SD	133.4 ± 240.1	128.9 ± 146.9	0.90
CRP, mg/dL, mean ± SD	2.4 ± 4.7	3.3 ± 4.0	0.27
CA19-9, U/ml, mean ± SD	561.6 ± 2475.8	6719.1 ± 36,162.6	0.21
M30, U/L, mean ± SD	212.5 ± 133.5	464.2 ± 305.7	< 0.01
MIC-1, 10^–2^ ng/ml, mean ± SD	228.2 ± 149.2	379.0 ± 204.5	< 0.01

**Fig. 3:**
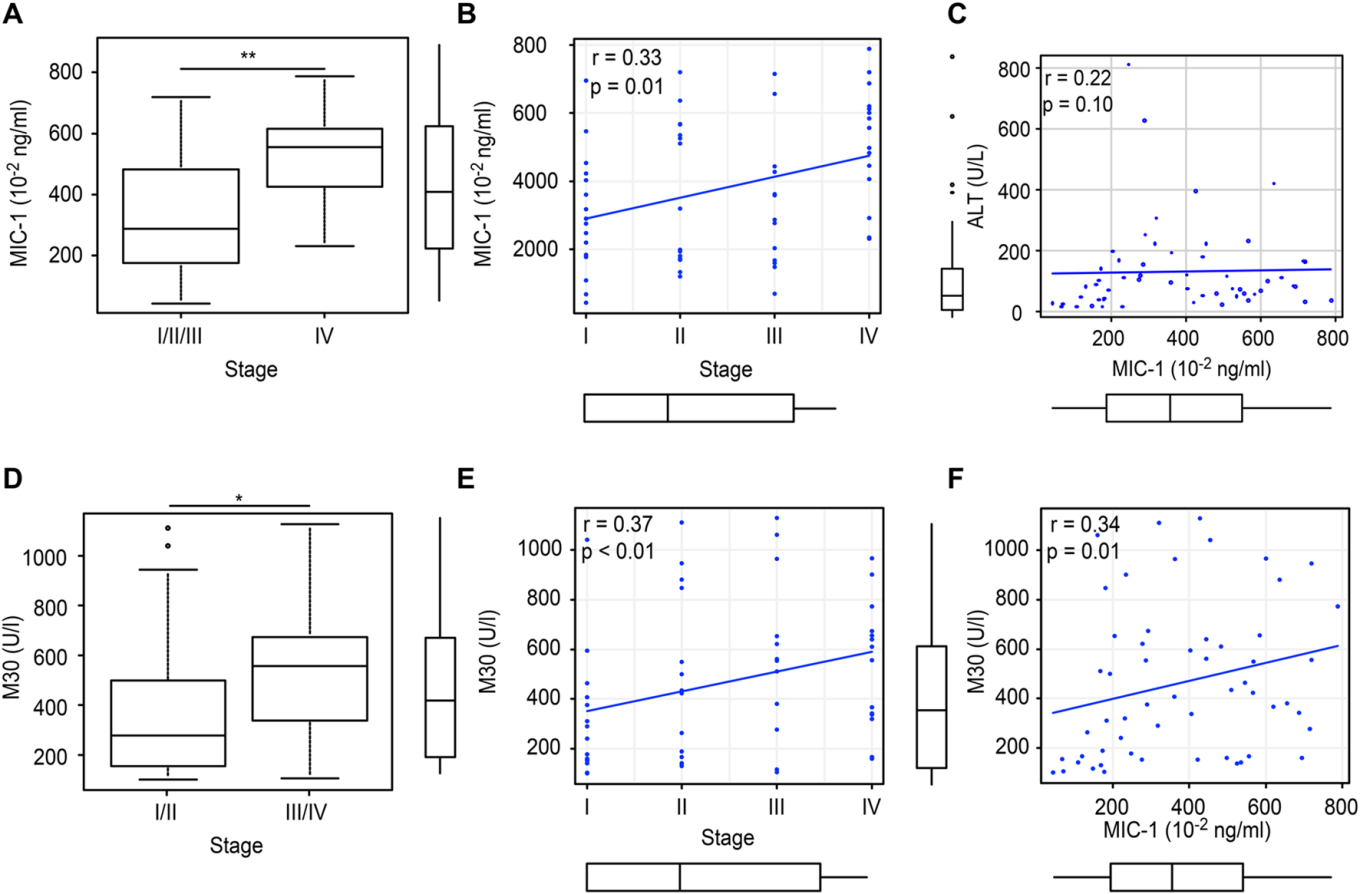
Correlation between serum MIC-1 levels and several clinical parameters in patients with BTC. **A** Serum MIC-1 levels were significantly higher in patients with stage IV BTC than in patients with stage I/II/III BTC (526.6 (231.1–788.4) vs. 288.1 (42.7–720.2) × 10^–2^ ng/ml, *P* < 0.01). **B** Serum MIC-1 levels and UICC stage showed a significant positive correlation. **C** Serum MIC-1 and ALT levels did not show a significant correlation. **D** Serum M30 levels were significantly higher in patients with stage III/IV BTC than in patients with stage I/II BTC (558.0 (105.4–1128.2) vs. 277.0 (100.6–1110.6) U/L, *p* = 0.015). **E** Serum M30 levels and the UICC stage showed a significant positive correlation. **F** Serum M30 and MIC-1 levels showed a significant positive correlation. * *P* < 0.05 and ** *P* < 0.01

In patients with BTC, significantly higher serum MIC-1 levels were detected in patients with stage IV tumours than in patients with stage I/II/III tumours (526.6 (231.1–788.4) vs. 288.1 (42.7–720.2) × 10^–2^ ng/ml; *p* < 0.01) (Fig. 3A), and serum MIC-1 levels showed a significant positive correlation with the UICC stage (r = 0.33; *p* = 0.01) (Fig. 3B). MIC-1 levels did not show a significant correlation with ALT levels (r = 0.22, *p* = 0.10) (Fig. 3C). Moreover, significantly higher serum M30 levels were observed in patients with stage III/IV BTC than in those with stage I/II disease (558.0 (105.4–1128.2) vs. 277.0 (100.6–1110.6) U/l; *p* = 0.015) (Fig. 3D). Furthermore, significant positive correlations were observed between serum M30 levels and both the UICC stage and serum MIC-1 levels (M30 and UICC stage: r = 0.37, *p* < 0.01; M30 and MIC-1: r = 0.34, *p* = 0.01) (Fig. 3E, F).

**Fig. 4:**
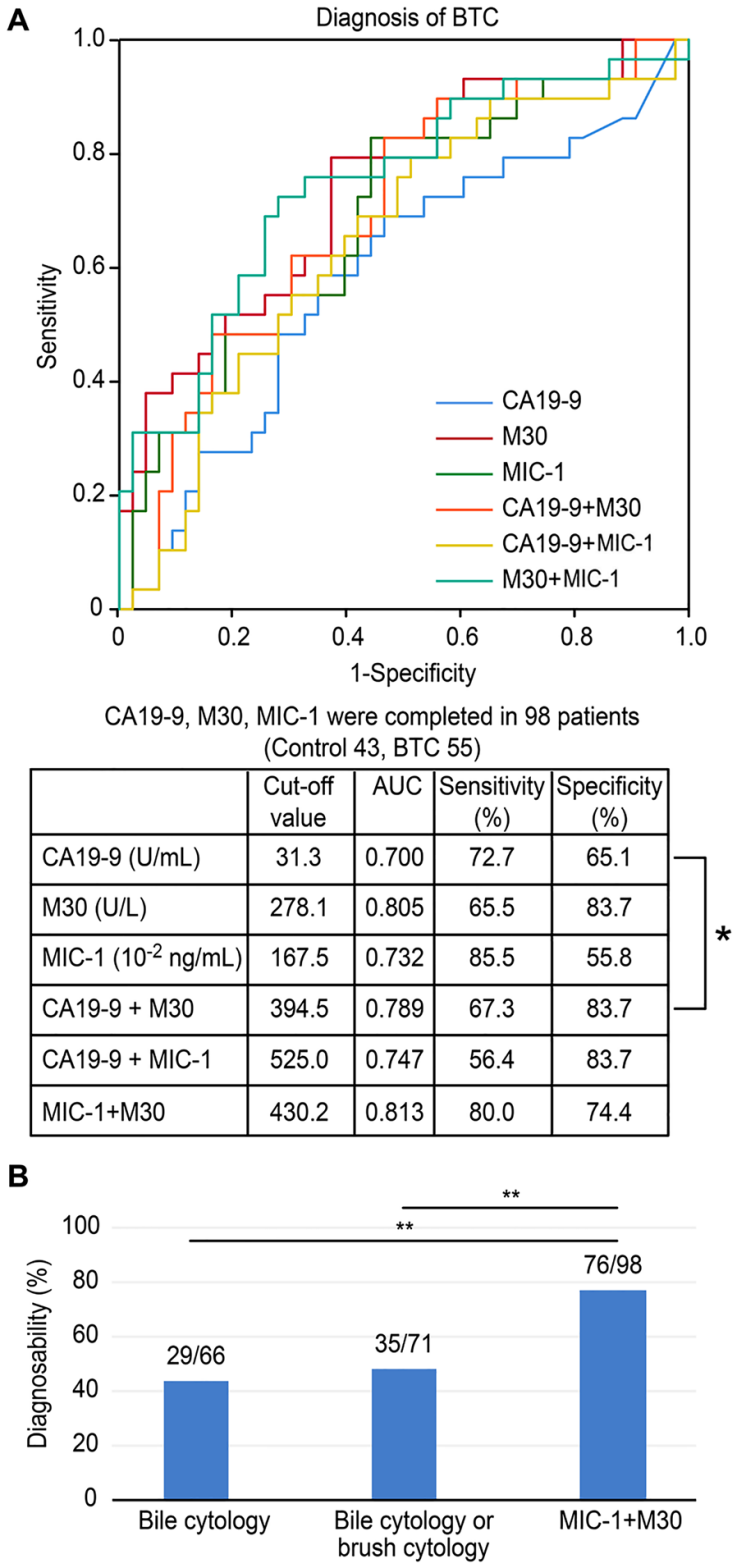
Ability to diagnose BTC using serum markers. **A** The AUCs of M30 and MIC-1 were higher than that of CA19-9. The AUC of the combination of CA19-9 and M30 was significantly higher than that of CA19-9. Furthermore, the combination of MIC-1 and M30 resulted in the highest AUC. **B** The ability to diagnose BTC was significantly greater using a combination of MIC-1 and M30 than using bile cytology or biliary brush cytology. * *P* < 0.05, ** *P* < 0.01

ROC curves were generated to evaluate the ability of various serum markers to diagnose BTC, and the area under the curve (AUC) for M30 and MIC-1 were higher than the corresponding value for CA19-9 (M30: AUC: 0.805, sensitivity: 65.5%, specificity: 83.7%; MIC-1: AUC: 0.732, sensitivity: 85.5%, specificity: 55.8%; CA19-9: AUC: 0.7, sensitivity: 72.7%, specificity: 65.1%) (Fig. 4A). The AUC of the combination of CA19-9 + M30 (AUC: 0.789, sensitivity: 67.3%, specificity: 83.7%) was significantly higher than that of CA19-9 (described above) (*P* value < 0.05). Additionally, the combination of MIC-1 and M30 had the highest AUC (AUC: 0.813, sensitivity: 80.0%, specificity: 74.4%). Bile cytology was performed in 66 patients. Biliary brush cytology was performed in 13 patients. The ability to diagnose BTC was significantly greater using the combination of MIC-1 and M30 (cut-off value: 430.2) than using bile cytology or biliary brush cytology (combination of MIC-1 and M30: 77.6%; bile cytology: 43.9%; bile cytology or brush cytology: 49.3%) (Fig. 4B).

Table 2 shows serum marker levels in patients with early BTC (stage I/II, n = 30). Serum M30 and MIC-1 levels were significantly higher in patients with early BTC than in control subjects (M30: 386.3 ± 300.6 vs. 212.5 ± 133.5 U/ml, *p* < 0.01; MIC-1: 338.7 ± 196.7 vs. 228.2 ± 149.2 × 10^–2^ ng/ml, *p* < 0.01).

**Table 2:** Comparison of clinical and demographic characteristics and serum markers in patients with benign disease (control) or early BTC

	Control	Early BTC	*P* value
N	62	30	
Age, y, mean ± SD	72.3 ± 11.8	73.3 ± 9.0	0.70
Male/female	38/24	24/6	0.12
The location of BTC			
Intrahepatic		1	
Peri-hilar		12	
Extrahepatic		17	
UICC stage			
I		16	
II		14	
AST, U/L, mean ± SD	115.7 ± 203.0	137.9 ± 259.4	0.69
ALT, U/L, mean ± SD	133.4 ± 240.1	144.5 ± 184.6	0.84
CRP, mg/dL, mean ± SD	2.4 ± 4.7	3.2 ± 4.4	0.40
CA19-9, U/ml, mean ± SD	561.6 ± 2475.8	479.1 ± 1935.6	0.88
M30, U/L, mean ± SD	212.5 ± 133.5	386.3 ± 300.6	< 0.01
MIC-1, 10^–2^ ng/ml, mean ± SD	228.2 ± 149.2	338.7 ± 196.7	< 0.01

**Fig. 5:**
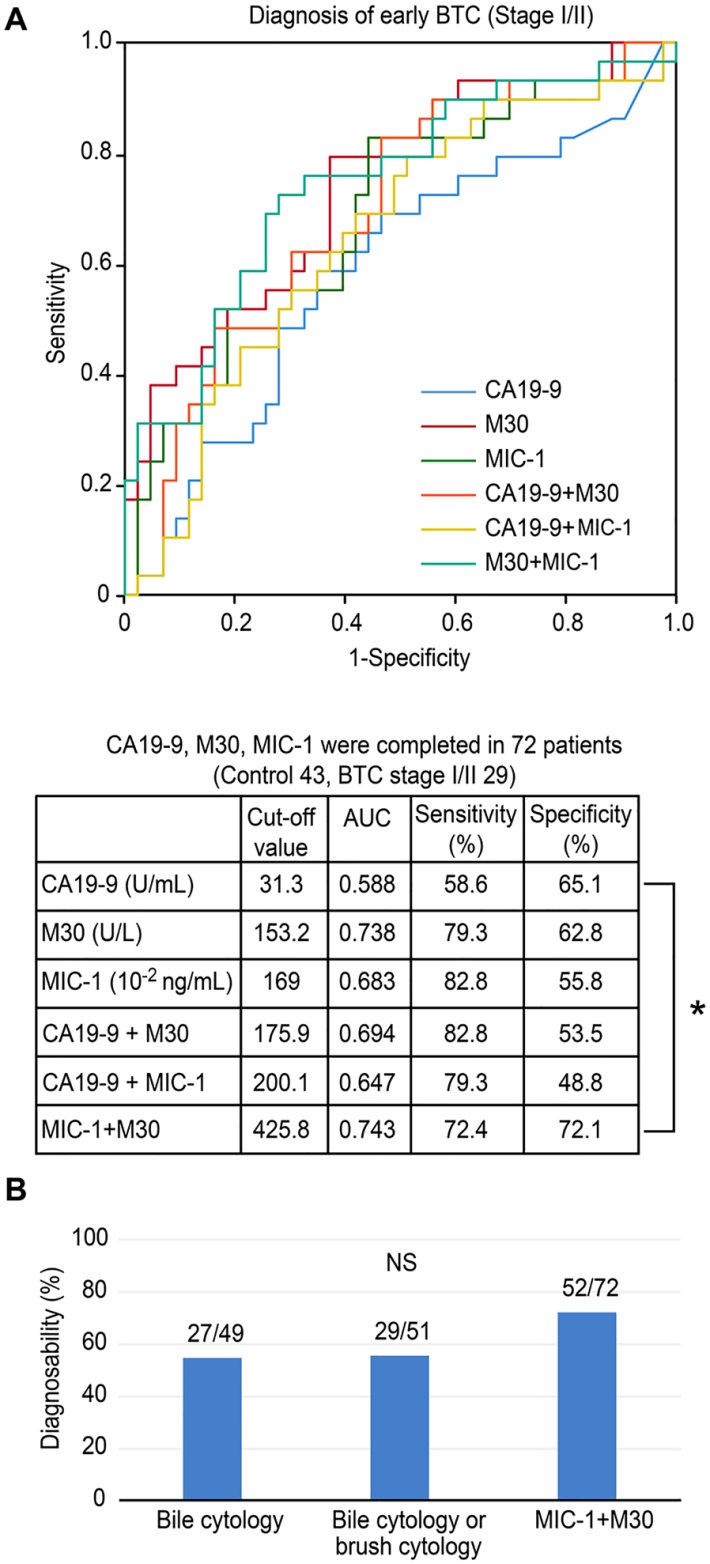
Ability to diagnose early BTC using serum markers. **A** The AUCs of MIC-1 and M30 were higher than that of CA19-9. The AUC was highest for the combination of MIC-1 and M30, indicating a greater ability to diagnose early BTC. The AUC of the combination of MIC-1 and M30 was significantly higher than that of CA19-9 (*P* value < 0.05). **B** Although the difference was not significant, the ability to diagnose BTC was improved using the combination of MIC-1 and M30 levels compared with biliary cytology or brush cytology. * *P* < 0.05

The ability of these markers to diagnose early BTC was similar to that of all-stage BTC (Fig. 5A). The AUC was highest for the combination of MIC-1 and M30 (AUC: 0.743, sensitivity: 72.4%, specificity: 72.1%), and the AUC of the combination of MIC-1 and M30 was significantly higher than that of CA19-9 (AUC: 0.588, sensitivity 58.6%, specificity 65.1%) (*P* value < 0.05). The combination of M30 and MIC-1 was better able to diagnose early BTC than bile cytology or brush cytology, although a significant difference was not observed (Fig. 5B).

**Fig. 6:**
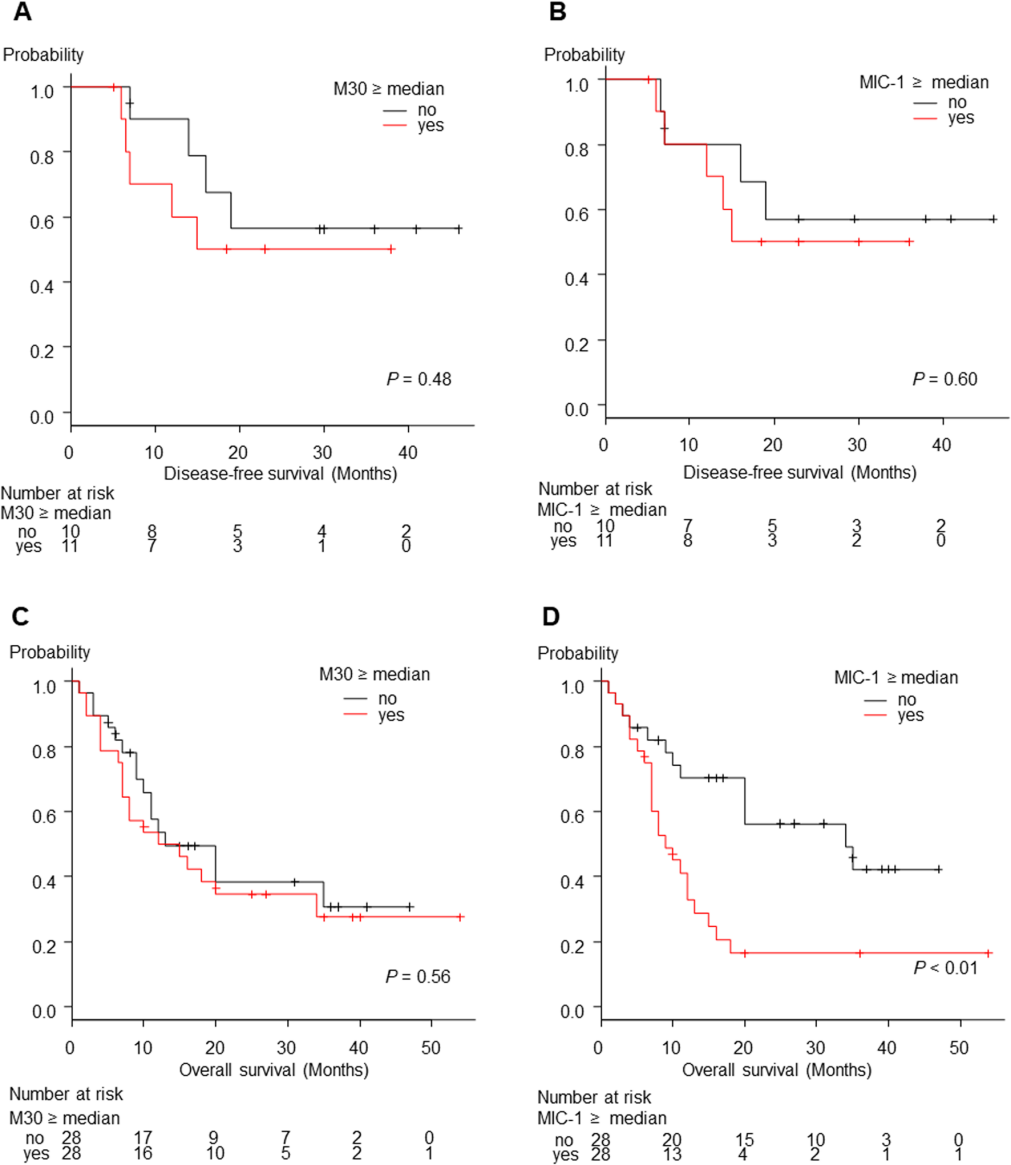
DFS, OS of BTC patients evaluated based on M30 or MIC-1. **A** The DFS was not significantly different between the patients with serum M30 ≥ the median and the patients with serum M30 < the median. **B** The DFS was not significantly different between the patients with serum MIC-1 ≥ the median and the patients with serum MIC-1 < the median. **C** The OS was not significantly different between the patients with serum M30 ≥ the median and the patients with serum M30 < the median. **D** The OS was significantly longer in the patients with serum MIC-1 < the median than in the patients with serum MIC-1 ≥ the median

DFS was not significantly different between the patients with serum M30 ≥ the median and the patients with serum M30 < the median (*P* value = 0.48 Fig. 6A). DFS was not significantly different between the patients with serum MIC-1 ≥ the median and the patients with serum MIC-1 < the median (*P* value = 0.60, Fig. 6B). OS was not significantly different between the patients with serum M30 ≥ the median and the patients with serum M30 < the median (*P* value = 0.56, Fig. 6C). However, OS was significantly longer in the patients with serum MIC-1 < the median than in the patients with serum MIC-1 ≥ the median (*P* value = 0.01, Fig. 6D).

## Discussion

The present study produce the results described below. 1. MIC-1 expression was observed in not only BTC cells but also normal bile duct epithelial cells. However, the intensity of MIC-1 immunostaining detected in the TMA was higher in BTC tissues than in normal tissues. Furthermore, MIC-1 expression was stronger in BTC cells than in normal bile duct epithelial cells, based on western blot and RT–PCR data. On the other hand, MIC-1 expression was detected in normal bile duct epithelial cells using western blotting and RT–PCR. Consequently, the MIC-1 expression levels observed in normal bile duct epithelial cells from surgical specimens were consistent with the western blotting and RT–PCR results. 2. MIC-1 suppressed BTC cell apoptosis and accelerated BTC proliferation and invasion; furthermore, MIC-1 inhibited cellular sensitivity to gemcitabine. 3. The serum levels of MIC-1 and the apoptosis marker M30 were significantly elevated in patients with BTC (all-stage and early BTC) compared with control subjects. Furthermore, serum MIC-1 levels correlated with the tumour stage and serum M30 levels. The combination of MIC-1 and M30 was an efficient diagnostic biomarker for BTC and early BTC. 4. BTC patients with high serum MIC-1 showed poor prognosis.

The function of MIC-1 depends on the tumour location. A summary of reports on the effect of MIC-1 on several cancers is provided in Table 3 [24, 32, 40–65]; however, the relationship between MIC-1 and BTC is unknown. In the present study, MIC-1 suppressed BTC cell apoptosis; therefore, we posit that MIC-1 promotes tumour progression. HuCCT-1 cell proliferation was stimulated by 6.25–50 ng/ml MIC-1 but not by 100–200 ng/ml MIC-1. Previous studies have used several concentrations of MIC-1 in cell proliferation assays; one such report treated cells with 0–40 ng/ml MIC-1 and found that pancreatic cancer cell lines exposed to MIC-1 exhibited a greater increase in proliferation than control cells [23]. Another previous study used 200 ng/ml MIC-1 [32], and although MIC-1 was reported to contribute to the maintenance of breast cancer stem-like cells, a breast cancer cell line treated with this concentration of MIC-1 showed less proliferation than the control cells. Based on previous publications and reported physiological concentrations, 100–200 ng/ml MIC-1 might be too high to positively affect the proliferation of cell lines.

**Table 3:** Reported functions of MIC-1 in several cancer types

Cancer	Author, year	Experimental level	Function
Neck Oesophagus	Li et al*.,* 2020 [61]	In vitro	Increases the number of invasive cells
	Urakawa et al*.,* 2015 [40]	In vitro	Associated with cancer growth
	Dong et al*.,* 2020 [66]	In vitro In vivo	Induces invasion and metastasis
	Okamoto et al*.,* 2020 [65]	In vitro	Increases proliferation, migration, and invasion
Lung	Duan et al*.,* 2019 [41]	In vitro In vivo	Inhibits proliferation, migration, and invasion
Breast	Kim et al*.,* 2008 [42]	In vitro	Participates in malignant progression
	Sasahara et al*.,* 2017 [32]	In vitro	Maintains cancer stem cell properties
	Wang et al*.,* 2018 [43]	In vitro In vivo	Represses metastatic potential
	Huang et al*.,* 2019 [64]	In vitro In vivo	Facilitates cancer progression via the GDF15–AKT pathway
	Gkretsi et al*.,* 2020 [62]	In vitro	Suppresses tumour cell invasion
Liver	Wang et al*.,* 2017 [44]	In vitro	Increases viability, invasion, migration, and angiogenesis
	Xu et al*.,* 2017 [45]	In vitro	Promotes metastasis
Stomach	Lee et al*.,* 2003 [46]	In vitro	Contributes to cancer progression
	Jang et al*.,* 2004 [47]	In vitro	Promotes the apoptosis of gastric cancer cells
	Kim et al*.,* 2008 [42]	In vitro	Participates in malignant progression
	Han et al*.,* 2017 [48]	In vitro	Activates apoptosis
Pancreas	Guo et al*.,* 2021 [24]	In vitro In vivo	Promotes cancer progression
Prostate	Liu et al*.,* 2003 [49]	In vitro	Reduces cell adhesion and induces apoptosis
	Senapati et al*.,* 2010 [50]	In vitro In vivo	Induces metastasis
	Huang et al*.,* 2014 [51]	In vitro In vivo	Promotes cancer progression
	Husaini et al*.,* 2015 [52]	In vitro In vivo	Promotes local invasion and metastatic spread
	Zhang et al*.,* 2019 [53]	In vitro In vivo	Inhibits cell proliferation and induces apoptosis
	Huang et al*.,* 2020 [60]	In vitro In vivo	Increases IL-8 and IL-6 levels and promotes cancer progression
Blatter	Tsui et al*.,* 2015 [54]	In vitro In vivo	Inhibits cell proliferation, invasion and tumorigenesis
Uterus	Li et al*.,* 2018 [55]	In vitro	Enhances cervical cancer cell proliferation
Ovary	Griner et al*.,* 2013 [56]	In vitro	Promotes cancer cell growth
Colon	Baek et al*.,* 2001 [57]	In vitro	Reduces tumorigenicity
	Li et al*.,* 2016 [58]	In vitro In vivo	Promotes the epithelial–mesenchymal transition and metastasis
	Zheng et al*.,* 2020 [63]	In vitro	Induces metastasis
Skin	Boyle et al*.,* 2009 [59]	In vitro In vivo	Facilitates the development of more aggressive melanoma

MIC-1 promoted cancer growth by suppressing the apoptosis of BTC cells. Thus, therapeutic agents targeting MIC-1 are a potential approach for treating BTC. A report described that MIC-1 is expressed after bile duct injury and may regulate bile duct proliferation and biliary tumour formation [67]. However, the detailed mechanism by which MIC-1 suppresses BTC cell apoptosis is unknown. In previous studies, commonalities between the mechanism of BTC progression and the protumorigenic effects of MIC-1 include increases in the activities of the JAK–STAT3 and PI3K–AKT signalling pathways. In a study of the relationship between MIC-1 and tumour progression, MIC-1 was reported to induce tumour progression via STAT3 activation [68], and STAT-3 signalling was shown to prevent tumour cell apoptosis [69]. In addition, MIC-1 activates the PI3K–AKT signalling pathway and reduces apoptosis [70]. Regarding the mechanism of BTC progression, IL-6 was reported to activate the JAK–STAT3 pathway, which promotes tumour cell proliferation and invasion [71, 72]. In addition, activation of the PI3K–AKT pathway was reported to induce BTC progression [73–75]. In the future, the mechanism by which MIC-1 affects BTC progression may be clarified by investigating cytokines and cytokine-related intracellular signalling pathways.

In the present study, the serum MIC-1 level was a useful diagnostic marker of BTC, and elevated serum MIC-1 levels were also observed in patients with BTC and reflected tumour progression. Moreover, OS was significantly shorter in BTC patients with high serum MIC-1. Therefore, the serum MIC-1 level has potential as a useful prognostic biomarker of BTC. In addition, the combination of serum MIC-1 levels and serum levels of the apoptosis marker M30 was effective at diagnosing BTC. As described above, a previous study reported the efficacy of biliary MIC-1 levels in diagnosing individuals with BTC (cut-off value: 58.2 × 10^–2^ ng/ml, AUC 0.77, sensitivity 71.4%, specificity 82.8%) [18]; however, this method is limited by the invasive nature of measuring biliary MIC-1 levels. The application of serum MIC-1 and M30 levels overcomes this limitation and was superior to the diagnostic ability of biliary MIC-1 levels (the combination of serum MIC-1 and M30 levels for diagnosing BTC: AUC 0.813, sensitivity 80%, specificity 74.4%). Additionally, the serum MIC-1 level was positively correlated with the serum M30 level, a finding that differed from the cell culture experiment findings. One potential explanation for this discrepancy might be that MIC-1 increases tumour volume, and consequently, the apoptosis of tumour cells increases. The serum M30 level properly reflects tumour size [76]; therefore, the serum M30 level increases with cancer progression. Notably, the M30 level showed a positive correlation with the BTC stage (Fig. 3D; r = 0.37; P < 0.01, Spearman’s rank correlation coefficient).

The present study has several limitations. First, this study included a relatively small sample size and employed a single-centre design. However, the difference in serum MIC-1 levels between the two groups was 150.8 × 10^–2^ ng/ml, and the standard deviation for the comparison between two groups was 191.7 × 10^–2^ ng/ml. Thus, the total sample size necessary to achieve an α error of 5% and a β value of 0.2 was 52 cases. When serum MIC-1 levels were determined to be the main outcome, the minimum number of necessary cases was collected. Second, MIC-1 immunostaining was performed on surgical specimens from only two patients; then, MIC-1 expression was determined using western blotting and RT–PCR and compared between a normal bile duct epithelial cell line and BTC cell lines. Third, the diagnostic specificity of serum MIC-1 levels for BTC was insufficient. However, serum MIC-1 levels are higher in patients with pancreaticobiliary cancer than in patients with other cancers or pancreaticobiliary inflammatory disease [15, 17]. In addition, the specificity was improved by combination with a serum apoptosis biomarker.

## Conclusions

In summary, previous reports have analysed MIC-1 in several types of cancer. However, serum MIC-1 levels were shown to be elevated to a greater extent in patients with pancreaticobiliary cancer than in patients with other types of cancer. Although MIC-1 and pancreatic cancer have been the focus of several previous studies, the effect and efficacy of MIC-1 have not been investigated in BTC. We previously reported the efficacy of evaluating biliary MIC-1 levels in diagnosing BTC. However, bile collection is an invasive procedure. The novelty of this study is the documentation of the effect of MIC-1 on BTC and the establishment of a new, noninvasive diagnostic method for BTC. Moreover, BTC patients with high serum MIC-1 had a poor prognosis. In conclusion, MIC-1 suppresses BTC cell apoptosis and promotes BTC progression. Additionally, the serum MIC-1 level reflects BTC progression, apoptosis, and prognosis of BTC patients. The ability to diagnose BTC is improved using the combination of serum MIC-1 and M30 levels, which is also useful for diagnosing early BTC. Therefore, MIC-1 might be a useful biomarker, prognostic marker, and therapeutic target in BTC.

## Data Availability

The datasets generated and/or analysed during the current study are available from the corresponding author upon reasonable request.

## Abbreviations

BTC: Biliary tract cancer
MIC-1: Macrophage inhibitory cytokine-1
TMA: Tissue microarray
RT–PCR: Reverse transcription PCR
FBS: Foetal bovine serum
CCK-8: Cell counting Kit-8
CBD: Common bile duct
AIP: Autoimmune pancreatitis
IPMN: Intraductal papillary neoplasm
DFS: Disease-free survival
OS: Overall survival
ROC: Receiver operating characteristic
UICC: Union for International Cancer Control
GEM: Gemcitabine
SD: Standard deviation
AUC: Area under the curve
NS: Not significant

## References

[1] Ishihara S, Horiguchi A, Miyakawa S, Endo I, Miyazaki M, Takada T (2016). Biliary tract cancer registry in Japan from 2008 to 2013. J Hepatobiliary Pancreat Sci.

[2] Miyakawa S, Ishihara S, Horiguchi A, Takada T, Miyazaki M, Nagakawa T (2009). Biliary tract cancer treatment: 5,584 results from the biliary tract cancer statistics registry from 1998 to 2004 in Japan. J Hepatobiliary Pancreat Surg.

[3] Okusaka T, Nakachi K, Fukutomi A, Mizuno N, Ohkawa S, Funakoshi A (2010). Gemcitabine alone or in combination with cisplatin in patients with biliary tract cancer: a comparative multicentre study in Japan. Br J Cancer.

[4] Valle J, Wasan H, Palmer DH, Cunningham D, Anthoney A, Maraveyas A (2010). Cisplatin plus gemcitabine versus gemcitabine for biliary tract cancer. N Engl J Med.

[5] Morizane C, Okusaka T, Mizusawa J, Takashima A, Ueno M, Ikeda M (2013). Randomized phase II study of gemcitabine plus S-1 versus S-1 in advanced biliary tract cancer: a Japan Clinical Oncology Group trial (JCOG 0805). Cancer Sci.

[6] Valle JW, Wasan H, Lopes A, Backen AC, Palmer DH, Morris K (2015). Cediranib or placebo in combination with cisplatin and gemcitabine chemotherapy for patients with advanced biliary tract cancer (ABC-03): a randomised phase 2 trial. Lancet Oncol.

[7] Morizane C, Okusaka T, Mizusawa J, Katayama H, Ueno M, Ikeda M (2019). Combination gemcitabine plus S-1 versus gemcitabine plus cisplatin for advanced/recurrent biliary tract cancer: The FUGA-BT (JCOG1113) randomized phase III clinical trial. Ann Oncol.

[8] Miyazaki M, Yoshitomi H, Miyakawa S, Uesaka K, Unno M, Endo I (2015). Clinical practice guidelines for the management of biliary tract cancers 2015: the 2nd English edition. J Hepatobiliary Pancreat Sci.

[9] Wang YF, Feng FL, Zhao XH, Ye ZX, Zeng HP, Li Z (2014). Combined detection tumor markers for diagnosis and prognosis of gallbladder cancer. World J Gastroenterol.

[10] Liang B, Zhong L, He Q, Wang S, Pan Z, Wang T (2015). Diagnostic accuracy of serum CA19-9 in patients with cholangiocarcinoma: a systematic review and meta-analysis. Med Sci Monit.

[11] Sheen-Chen SM, Sun CK, Liu YW, Eng HL, Ko SF, Kuo CH (2007). Extremely elevated CA19-9 in acute cholangitis. Dig Dis Sci.

[12] Marrelli D, Caruso S, Pedrazzani C, Neri A, Fernandes E, Marini M (2009). CA19-9 serum levels in obstructive jaundice: clinical value in benign and malignant conditions. Am J Surg.

[13] Bootcov MR, Bauskin AR, Valenzuela SM, Moore AG, Bansal M, He XY (1997). MIC-1, a novel macrophage inhibitory cytokine, is a divergent member of the TGF-beta superfamily. Proc Natl Acad Sci U S A.

[14] Bauskin AR, Brown DA, Kuffner T, Johnen H, Luo XW, Hunter M (2006). Role of macrophage inhibitory cytokine-1 in tumorigenesis and diagnosis of cancer. Cancer Res.

[15] Koopmann J, Buckhaults P, Brown DA, Zahurak ML, Sato N, Fukushima N (2004). Serum macrophage inhibitory cytokine 1 as a marker of pancreatic and other periampullary cancers. Clin Cancer Res.

[16] Koopmann J, Rosenzweig CN, Zhang Z, Canto MI, Brown DA, Hunter M (2006). Serum markers in patients with resectable pancreatic adenocarcinoma: Macrophage inhibitory cytokine 1 versus CA19-9. Clin Cancer Res.

[17] Wang X, Li Y, Tian H, Qi J, Li M, Fu C (2014). Macrophage inhibitory cytokine 1 (MIC-1/GDF15) as a novel diagnostic serum biomarker in pancreatic ductal adenocarcinoma. BMC Cancer.

[18] Sugimoto M, Takagi T, Konno N, Suzuki R, Asama H, Watanabe K (2017). The efficacy of biliary and serum macrophage inhibitory cytokine-1 for diagnosing biliary tract cancer. Sci Rep.

[19] Babic A, Schnure N, Neupane NP, Zaman MM, Rifai N, Welch MW (2018). Plasma inflammatory cytokines and survival of pancreatic cancer patients. Clin Transl Gastroenterol.

[20] Hogendorf P, Durczyński A, Skulimowski A, Kumor A, Poznańska G, Strzelczyk J (2018). Growth differentiation factor (GDF-15) concentration combined with Ca125 levels in serum is superior to commonly used cancer biomarkers in differentiation of pancreatic mass. Cancer Biomark.

[21] Yang Y, Yan S, Tian H, Bao Y (2018). Macrophage inhibitory cytokine-1 versus carbohydrate antigen 19–9 as a biomarker for diagnosis of pancreatic cancer: a PRISMA-compliant meta-analysis of diagnostic accuracy studies. Medicine (Baltimore).

[22] O'Neill RS, Emmanuel S, Williams D, Stoita A (2020). Macrophage inhibitory cytokine-1/growth differentiation factor-15 in premalignant and neoplastic tumours in a high-risk pancreatic cancer cohort. World J Gastroenterol.

[23] Zhao Z, Zhang J, Yin L, Yang J, Zheng Y, Zhang M (2020). Upregulated GDF-15 expression facilitates pancreatic ductal adenocarcinoma progression through orphan receptor GFRAL. Aging (Albany NY).

[24] Guo F, Zhou Y, Guo H, Ren D, Jin X, Wu H (2021). NR5A2 transcriptional activation by BRD4 promotes pancreatic cancer progression by upregulating GDF15. Cell Death Discov.

[25] Ozkan H, Demirbaş S, Ibiş M, Akbal E, Köklü S (2011). Diagnostic validity of serum macrophage inhibitor cytokine and tissue polypeptide-specific antigen in pancreatobiliary diseases. Pancreatology.

[26] Kirkegaard T, Edwards J, Tovey S, McGlynn LM, Krishna SN, Mukherjee R (2006). Observer variation in immunohistochemical analysis of protein expression, time for a change?. Histopathology.

[27] Suzuki R, Okubo Y, Takagi T, Sugimoto M, Sato Y, Irie H (2022). The complement C3a–C3a receptor axis regulates epithelial-to-mesenchymal transition by activating the ERK pathway in pancreatic ductal adenocarcinoma. Anticancer Res.

[28] Maruyama M, Kobayashi N, Westerman KA, Sakaguchi M, Allain JE, Totsugawa T (2004). Establishment of a highly differentiated immortalized human cholangiocyte cell line with SV40T and hTERT. Transplantation.

[29] Miyagiwa M, Ichida T, Tokiwa T, Sato J, Sasaki H (1989). A new human cholangiocellular carcinoma cell line (HuCC-T1) producing carbohydrate antigen 19/9 in serum-free medium. In Vitro Cell Dev Biol.

[30] Saijyo S, Kudo T, Suzuki M, Katayose Y, Shinoda M, Muto T (1995). Establishment of a new extrahepatic bile duct carcinoma cell line, TFK-1. Tohoku J Exp Med.

[31] Nagathihalli NS, Castellanos JA, VanSaun MN, Dai X, Ambrose M, Guo Q (2016). Pancreatic stellate cell secreted IL-6 stimulates STAT3 dependent invasiveness of pancreatic intraepithelial neoplasia and cancer cells. Oncotarget.

[32] Sasahara A, Tominaga K, Nishimura T, Yano M, Kiyokawa E, Noguchi M (2017). An autocrine/paracrine circuit of growth differentiation factor (GDF) 15 has a role for maintenance of breast cancer stem-like cells. Oncotarget.

[33] Zhao Z, Zhang J, Yin L, Yang J, Zheng Y, Zhang M (2020). Upregulated GDF-15 expression facilitates pancreatic ductal adenocarcinoma progression through orphan receptor GFRAL. Aging (Albany NY).

[34] Hayashi M, Abe K, Fujita M, Okai K, Takahashi A, Nozawa Y (2018). Serum levels of a cell death biomarker predict the development of cirrhosis-related conditions in primary biliary cholangitis. Med Mol Morphol.

[35] Sugimoto M, Abe K, Hayashi M, Takagi T, Suzuki R, Konno N (2018). The efficacy of serum cell death biomarkers for diagnosing biliary tract cancer. Sci Rep.

[36] Shimosegawa T, Chari ST, Frulloni L, Kamisawa T, Kawa S, Mino-Kenudson M (2011). International consensus diagnostic criteria for autoimmune pancreatitis: Guidelines of the International association of pancreatology. Pancreas.

[37] Tanaka M, Fernández-Del Castillo C, Kamisawa T, Jang JY, Levy P, Ohtsuka T (2017). Revisions of international consensus Fukuoka guidelines for the management of IPMN of the pancreas. Pancreatology.

[38] Brierley JD, Gospodarowicz MK, Wittekind C (2017). TNM-classification of malignant tumours.

[39] Kanda Y (2013). Investigation of the freely available easy-to-use software 'EZR' for medical statistics. Bone Marrow Transplant.

[40] Urakawa N, Utsunomiya S, Nishio M, Shigeoka M, Takase N, Arai N (2015). GDF15 derived from both tumor-associated macrophages and esophageal squamous cell carcinomas contributes to tumor progression via Akt and Erk pathways. Lab Invest.

[41] Duan L, Pang HL, Chen WJ, Shen WW, Cao PP, Wang SM (2019). The role of GDF15 in bone metastasis of lung adenocarcinoma cells. Oncol Rep.

[42] Kim KK, Lee JJ, Yang Y, You KH, Lee JH (2008). Macrophage inhibitory cytokine-1 activates AKT and ERK-1/2 via the transactivation of ErbB2 in human breast and gastric cancer cells. Carcinogenesis.

[43] Wang T, Mao B, Cheng C, Zou Z, Gao J, Yang Y (2018). YAP promotes breast cancer metastasis by repressing growth differentiation factor-15. Biochim Biophys Acta Mol Basis Dis.

[44] Wang L, Liu Y, Li W, Song Z (2017). Growth differentiation factor 15 promotes cell viability, invasion, migration, and angiogenesis in human liver carcinoma cell line HepG2. Clin Res Hepatol Gastroenterol.

[45] Xu Q, Xu HX, Li JP, Wang S, Fu Z, Jia J (2017). Growth differentiation factor 15 induces growth and metastasis of human liver cancer stem-like cells via AKT/GSK-3beta/beta-catenin signaling. Oncotarget.

[46] Lee DH, Yang Y, Lee SJ, Kim KY, Koo TH, Shin SM (2003). Macrophage inhibitory cytokine-1 induces the invasiveness of gastric cancer cells by up-regulating the urokinase-type plasminogen activator system. Cancer Res.

[47] Jang TJ, Kang HJ, Kim JR, Yang CH (2004). Non-steroidal anti-inflammatory drug activated gene (NAG-1) expression is closely related to death receptor-4 and -5 induction, which may explain sulindac sulfide induced gastric cancer cell apoptosis. Carcinogenesis.

[48] Han M, Dai D, Yousafzai NA, Wang F, Wang H, Zhou Q (2017). CXXC4 activates apoptosis through up-regulating GDF15 in gastric cancer. Oncotarget.

[49] Liu T, Bauskin AR, Zaunders J, Brown DA, Pankhurst S, Russell PJ (2003). Macrophage inhibitory cytokine 1 reduces cell adhesion and induces apoptosis in prostate cancer cells. Cancer Res.

[50] Senapati S, Rachagani S, Chaudhary K, Johansson SL, Singh RK, Batra SK (2010). Overexpression of macrophage inhibitory cytokine-1 induces metastasis of human prostate cancer cells through the FAK-RhoA signaling pathway. Oncogene.

[51] Huang M, Narita S, Inoue T, Tsuchiya N, Satoh S, Nanjo H (2014). Diet-induced macrophage inhibitory cytokine 1 promotes prostate cancer progression. Endocr Relat Cancer.

[52] Husaini Y, Lockwood GP, Nguyen TV, Tsai VW, Mohammad MG, Russell PJ (2015). Macrophage inhibitory cytokine-1 (MIC-1/GDF15) gene deletion promotes cancer growth in TRAMP prostate cancer prone mice. PLoS ONE.

[53] Zhang W, Hu C, Wang X, Bai S, Cao S, Kobelski M (2019). Role of GDF15 in methylseleninic acid-mediated inhibition of cell proliferation and induction of apoptosis in prostate cancer cells. PLoS ONE.

[54] Tsui KH, Hsu SY, Chung LC, Lin YH, Feng TH, Lee TY (2015). Growth differentiation factor-15: a p53- and demethylation-upregulating gene represses cell proliferation, invasion, and tumorigenesis in bladder carcinoma cells. Sci Rep.

[55] Li S, Ma YM, Zheng PS, Zhang P (2018). GDF15 promotes the proliferation of cervical cancer cells by phosphorylating AKT1 and Erk1/2 through the receptor ErbB2. J Exp Clin Cancer Res.

[56] Griner SE, Joshi JP, Nahta R (2013). Growth differentiation factor 15 stimulates rapamycin-sensitive ovarian cancer cell growth and invasion. Biochem Pharmacol.

[57] Baek SJ, Kim KS, Nixon JB, Wilson LC, Eling TE (2001). Cyclooxygenase inhibitors regulate the expression of a TGF-beta superfamily member that has proapoptotic and antitumorigenic activities. Mol Pharmacol.

[58] Li C, Wang J, Kong J, Tang J, Wu Y, Xu E (2016). GDF15 promotes EMT and metastasis in colorectal cancer. Oncotarget.

[59] Boyle GM, Pedley J, Martyn AC, Banducci KJ, Strutton GM, Brown DA (2009). Macrophage inhibitory cytokine-1 is overexpressed in malignant melanoma and is associated with tumorigenicity. J Invest Dermatol.

[60] Huang M, Narita S, Koizumi A, Nara T, Numakura K, Satoh S (2021). Macrophage inhibitory cytokine-1 induced by a high-fat diet promotes prostate cancer progression by stimulating tumor-promoting cytokine production from tumor stromal cells. Cancer Commun (Lond).

[61] Li L, Zhang R, Yang H, Zhang D, Liu J, Li J (2020). GDF15 knockdown suppresses cervical cancer cell migration *in vitro* through the TGF-β/Smad2/3/Snail1 pathway. FEBS Open Bio.

[62] Gkretsi V, Stylianou A, Kalli M, Louca M, Voutouri C, Zaravinos A (2020). Silencing of growth differentiation factor-15 promotes breast cancer cell invasion by down-regulating focal adhesion genes. Anticancer Res.

[63] Zheng H, Wu Y, Guo T, Liu F, Xu Y, Cai S (2020). Hypoxia induces growth differentiation factor 15 to promote the metastasis of colorectal cancer via PERK-eIF2α signaling. Biomed Res Int.

[64] Huang JY, Wang YY, Lo S, Tseng LM, Chen DR, Wu YC (2019). Visfatin mediates malignant behaviors through adipose-derived stem cells intermediary in breast cancer. Cancers (Basel).

[65] Okamoto M, Koma YI, Kodama T, Nishio M, Shigeoka M, Yokozaki H (2020). Growth differentiation factor 15 promotes progression of esophageal squamous cell carcinoma via TGF-β type II receptor activation. Pathobiology.

[66] Dong G, Huang X, Jiang S, Ni L, Ma L, Zhu C (2020). SCAP Mediated GDF15-Induced Invasion and EMT of esophageal cancer. Front Oncol.

[67] Koniaris LG (2003). Induction of MIC-1/growth differentiation factor-15 following bile duct injury. J Gastrointest Surg.

[68] Kang YE, Kim JM, Lim MA, Lee SE, Yi S, Kim JT (2021). Growth differentiation factor 15 is a cancer cell-induced mitokine that primes thyroid cancer cells for invasiveness. Thyroid.

[69] Yan H, Guo BY, Zhang S (2016). Cancer-associated fibroblasts attenuate Cisplatin-induced apoptosis in ovarian cancer cells by promoting STAT3 signaling. Biochem Biophys Res Commun.

[70] Chen L, Yin Y, Liu G (2021). Metformin alleviates bevacizumab-induced vascular endothelial injury by up-regulating GDF15 and activating the PI3K/AKT/FOXO/PPARγ signaling pathway. Ann Transl Med.

[71] Huyen NT, Prachayasittikul V, Chan-On W (2016). Anoikis-resistant cholangiocarcinoma cells display aggressive characteristics and increase STAT3 activation. J Hepatobiliary Pancreat Sci.

[72] Yamanaka T, Harimoto N, Yokobori T, Muranushi R, Hoshino K, Hagiwara K (2020). Nintedanib inhibits intrahepatic cholangiocarcinoma aggressiveness via suppression of cytokines extracted from activated cancer-associated fibroblasts. Br J Cancer.

[73] Ikenoue T, Terakado Y, Nakagawa H, Hikiba Y, Fujii T, Matsubara D (2016). A novel mouse model of intrahepatic cholangiocarcinoma induced by liver-specific Kras activation and Pten deletion. Sci Rep.

[74] Liang S, Guo H, Ma K, Li X, Wu D, Wang Y (2021). A PLCB1-PI3K-AKT signaling axis activates EMT to promote cholangiocarcinoma progression. Cancer Res.

[75] Liu H, Liu C, Wang M, Sun D, Zhu P, Zhang P (2021). Tanshinone IIA affects the malignant growth of cholangiocarcinoma cells by inhibiting the PI3K-Akt-mTOR pathway. Sci Rep.

[76] Koelink PJ, Lamers CB, Hommes DW, Verspaget HW (2009). Circulating cell death products predict clinical outcome of colorectal cancer patients. BMC Cancer.

